# Supplementary material for: Deep learning workflow to support in-flight processing of digital aerial imagery for wildlife population surveys
Source: PLoS One. 2024 Apr 3;19(4):e0288121. doi: 10.1371/journal.pone.0288121 (PMC10990224; doi:10.1371/journal.pone.0288121)
Supplement: S1 Table — The table details the subdivision of the imagery dataset used in this study into a training, a validation, and a test dataset. The training and validation datasets consist of images from the Nantucket Shoals study area, while the test dataset consists of images from Lake Michigan. An additional 69 images from the Lake Michigan study area, independent from the test dataset are not listed in the table, were used for further performance evaluation of the binary classifier algorithm. (PDF) [file pone.0288121.s001.pdf]

Image Id	Study Area	Subdataset
56257	Nantucket Shoals	Training
56068	Nantucket Shoals	Training
58875	Nantucket Shoals	Training
56262	Nantucket Shoals	Training
50268	Nantucket Shoals	Training
58996	Nantucket Shoals	Training
54729	Nantucket Shoals	Training
50311	Nantucket Shoals	Training
50247	Nantucket Shoals	Training
54724	Nantucket Shoals	Training
59713	Nantucket Shoals	Training
58878	Nantucket Shoals	Training
58799	Nantucket Shoals	Training
58785	Nantucket Shoals	Training
59860	Nantucket Shoals	Training
57258	Nantucket Shoals	Training
56201	Nantucket Shoals	Training
51741	Nantucket Shoals	Training
59000	Nantucket Shoals	Training
58874	Nantucket Shoals	Training
50367	Nantucket Shoals	Training
58787	Nantucket Shoals	Training
59710	Nantucket Shoals	Training
51739	Nantucket Shoals	Training
59454	Nantucket Shoals	Training
58872	Nantucket Shoals	Training
56265	Nantucket Shoals	Training
59002	Nantucket Shoals	Training
50228	Nantucket Shoals	Training
50304	Nantucket Shoals	Training
56461	Nantucket Shoals	Training
50038	Nantucket Shoals	Training
58877	Nantucket Shoals	Training
56260	Nantucket Shoals	Training
50206	Nantucket Shoals	Training
56259	Nantucket Shoals	Training
56288	Nantucket Shoals	Training
50135	Nantucket Shoals	Training

Image Id	Study Area	Subdataset
50275	Nantucket Shoals	Validation
56153	Nantucket Shoals	Validation
58993	Nantucket Shoals	Validation
50232	Nantucket Shoals	Validation
49960	Nantucket Shoals	Validation
58871	Nantucket Shoals	Validation
58869	Nantucket Shoals	Validation
58798	Nantucket Shoals	Validation
51740	Nantucket Shoals	Validation
56284	Nantucket Shoals	Validation
58873	Nantucket Shoals	Validation
57171	Nantucket Shoals	Validation
49874	Nantucket Shoals	Validation
51742	Nantucket Shoals	Validation
50242	Nantucket Shoals	Validation
56258	Nantucket Shoals	Validation
50043	Nantucket Shoals	Validation
50341	Nantucket Shoals	Validation
54728	Nantucket Shoals	Validation
58887	Nantucket Shoals	Validation
39875	Lake Michigan	Test
39858	Lake Michigan	Test
39855	Lake Michigan	Test
39900	Lake Michigan	Test
39904	Lake Michigan	Test
39902	Lake Michigan	Test
39853	Lake Michigan	Test
39856	Lake Michigan	Test
39903	Lake Michigan	Test
