# Supplementary material for: Deep learning workflow to support in-flight processing of digital aerial imagery for wildlife population surveys
Source: PLoS One. 2024 Apr 3;19(4):e0288121. doi: 10.1371/journal.pone.0288121 (PMC10990224; doi:10.1371/journal.pone.0288121)
Supplement: S2 Table — An additional 69 images from the Lake Michigan study area, independent from the test dataset, were used together with 9 images from test dataset for further performance evaluation of the binary classifier algorithm. (PDF) [file pone.0288121.s002.pdf]

Image Id	Study Area	Subdataset
39831	Lake Michigan	Test
39832	Lake Michigan	Test
39833	Lake Michigan	Test
39834	Lake Michigan	Test
39835	Lake Michigan	Test
39836	Lake Michigan	Test
39837	Lake Michigan	Test
39838	Lake Michigan	Test
39839	Lake Michigan	Test
39840	Lake Michigan	Test
39841	Lake Michigan	Test
39842	Lake Michigan	Test
39843	Lake Michigan	Test
39844	Lake Michigan	Test
39845	Lake Michigan	Test
39846	Lake Michigan	Test
39849	Lake Michigan	Test
39850	Lake Michigan	Test
39851	Lake Michigan	Test
39852	Lake Michigan	Test
39854	Lake Michigan	Test
39857	Lake Michigan	Test
39859	Lake Michigan	Test
39860	Lake Michigan	Test
39861	Lake Michigan	Test
39862	Lake Michigan	Test
39863	Lake Michigan	Test
39864	Lake Michigan	Test
39865	Lake Michigan	Test
39866	Lake Michigan	Test
39867	Lake Michigan	Test
39868	Lake Michigan	Test
39869	Lake Michigan	Test
39870	Lake Michigan	Test
39871	Lake Michigan	Test

Image Id	Study Area	Subdataset
39872	Lake Michigan	Test
39873	Lake Michigan	Test
39874	Lake Michigan	Test
39876	Lake Michigan	Test
39877	Lake Michigan	Test
39878	Lake Michigan	Test
39879	Lake Michigan	Test
39880	Lake Michigan	Test
39881	Lake Michigan	Test
39882	Lake Michigan	Test
39883	Lake Michigan	Test
39884	Lake Michigan	Test
39885	Lake Michigan	Test
39886	Lake Michigan	Test
39887	Lake Michigan	Test
39888	Lake Michigan	Test
39889	Lake Michigan	Test
39890	Lake Michigan	Test
39891	Lake Michigan	Test
39892	Lake Michigan	Test
39893	Lake Michigan	Test
39894	Lake Michigan	Test
39895	Lake Michigan	Test
39896	Lake Michigan	Test
39897	Lake Michigan	Test
39901	Lake Michigan	Test
39905	Lake Michigan	Test
39906	Lake Michigan	Test
39907	Lake Michigan	Test
39908	Lake Michigan	Test
39909	Lake Michigan	Test
39910	Lake Michigan	Test
39911	Lake Michigan	Test
39912	Lake Michigan	Test
