# Supplementary material for: Deep learning workflow to support in-flight processing of digital aerial imagery for wildlife population surveys
Source: PLoS One. 2024 Apr 3;19(4):e0288121. doi: 10.1371/journal.pone.0288121 (PMC10990224; doi:10.1371/journal.pone.0288121)
Supplement: S1 File — The source information for the map data is covered by the Geogratis License Agreement for Unrestricted Use of Digital Data. No base map or other copyrighted material was used to create the map figure. (PDF) [file pone.0288121.s003.pdf]

```
Identification_Information:
  Citation:
    Citation_Information:
      Originator:
        Government of Canada, Natural Resources Canada, Mapping Information Branch, The
        Atlas of Canada
      Originator: Instituto Nacional de Estadística y Geografía
      Originator:
        United States Department of the Interior, U.S. Geological Survey, National Atlas of
        the United States
      Publication_Date: 2010
      Title: North American Atlas - Political Boundaries
      Geospatial_Data_Presentation_Form: Vector digital data
      Publication_Information:
        Publication_Place: Ottawa, Ontario, Canada
        Publisher: Government of Canada
      Online_Linkage:
        http://geogratis.gc.ca/download/frameworkdata/North_America_Atlas10M/boundaries/
      Publication_Information:
        Publication_Place: Aguascalientes, Aguascalientes, Mexico
        Publisher:
          Instituto Nacional de Estadística y Geografía (INEGI)
      Online_Linkage: http://antares.inegi.org.mx/atlas_norteamerica/
      Publication_Information:
        Publication_Place: Reston, Virginia, USA
        Publisher: U.S. Geological Survey, National Atlas of the United States
      Online_Linkage: http://nationalatlas.gov/atlasftp-na.html
      Publication_Information:
        Publication_Place: Montréal, Québec, Canada
        Publisher: Commission for Environmental Cooperation
      Online_Linkage: http://www.cec.org/naatlas/

  Description:
    Abstract:
      A joint venture involving the National Atlas programs in Canada (Natural Resources
      Canada), Mexico (Instituto Nacional de Estadística y Geografía), and the United States
      (U.S. Geological Survey), as well as the North American Commission for Environmental
      Cooperation, has led to the release (June 2004) of several new products: an updated paper
      map of North America, and its associated geospatial data sets and their metadata.  These
      data sets are available online from each of the partner countries for download.

      The North American Atlas data are standardized geospatial data sets at 1:10,000,000
      scale.  A variety of basic data layers (e.g. roads, railroads, populated places,
      political boundaries, hydrography, bathymetry, sea ice and glaciers) have been integrated
      so that their relative positions are correct.  This collection of data sets forms a base
      with which other North American thematic data may be integrated.  Any data outside of
      Canada, Mexico, and the United States of America included in the North American Atlas
      data sets is strictly to complete the context of the data.

      The North American Atlas - Political Boundaries data set shows political entities in
      North America as polygons representing jurisdictional areas, and as lines representing
      political boundaries, including International boundaries, Provincial boundaries, State or
      territory boundaries, and the International Date Line.

      This is a revised version of the 2006 data set.
```

Purpose:
The North American Atlas data are intended for geographic display and analysis at the national and continental level. These data should be displayed and analyzed at scales appropriate for 1:10,000,000-scale data. No responsibility is assumed by Natural Resources Canada, Instituto Nacional de Estadística y Geografía, National Atlas of the United States, or the Commission for Environmental Cooperation in the use of these data.

Supplemental_Information:
The Commission for Environmental Cooperation (CEC) is an international organization created by Canada, Mexico, and the United States of America under the North American Agreement on Environmental Cooperation (NAAEC). The CEC was established to address regional environmental concerns, help prevent potential trade and environmental conflicts, and to promote the effective enforcement of environmental law. The Agreement complements the environmental provisions of the North American Free Trade Agreement (NAFTA). Further information on the CEC is available from
http://www.cec.org/ or from
>Commission for Environmental Cooperation
>393, rue St-Jacques Ouest
>Bureau 200
>Montréal (Québec)
>H2Y 1N9 Canada
>
>Telephone: 1 514 350 4300
>Facsimile: 1 514 350 4314
>Electronic mail: info@cec.org
>

This data set is available in several formats, including Shapefile, layer package, and MXD. Included with each download are a readme file along with other files appropriate to the particular format, such as .avl and .lyr files. The data set is also available as a GeoPDF.

State boundaries in Mexico are included for illustration purposes. They were obtained from diverse sources and INEGI does not guarantee and takes no responsibility for their legal validity and/or accuracy.

These data are distributed in both geographic coordinates (through National Atlas of Canada, INEGI, and National Atlas of the United States), and a Lambert Azimuthal Equal Area projection (CEC). The following parameters are used for the Lambert Azimuthal Equal Area projection:
>Longitude_of_Projection_Center: -100.00
>Latitude_of_Projection_Center: 45.00
>False_Easting (metres): 0.0
>False_Northing (metres): 0.0
>Semi-major_Axis: 6370997.0
>Denominator_of_Flattening_Ratio: 1.0
>Ellipsoid_Name: Sphere
>Units: metres
>
The North American Environmental Atlas maintains this Political Boundaries data and all other base data sets in a geodatabase in a Lambert Azimuthal Equal Area projection.
Time_Period_of_Content:
Time_Period_Information:
Single_Date/Time:
Calendar_Date: 2010
Currentness_Reference: Date of most recent geometry edit

```
Status:
  Progress: Complete
  Maintenance_and_Update_Frequency: Irregular

Spatial_Domain:
  Bounding_Coordinates:
    West_Bounding_Coordinate: 152.0
    East_Bounding_Coordinate: 4.0
    North_Bounding_Coordinate: 90.0
    South_Bounding_Coordinate: -5.0

Keywords:
  Theme:
    Theme_Keyword_Thesaurus: GCMD Science keywords
    Theme_Keyword: Human Dimensions > Boundaries > Administrative Divisions
    Theme_Keyword: Human Dimensions > Boundaries > Political Divisions

  Theme:
    Theme_Keyword_Thesaurus: ISO 19115 Topic Category
    Theme_Keyword: boundaries
  Theme:
    Theme_Keyword_Thesaurus: None
    Theme_Keyword: Boundaries
    Theme_Keyword: Political boundaries
    Theme_Keyword: International boundaries
    Theme_Keyword: International data line
    Theme_Keyword: State boundaries
    Theme_Keyword: Provincial boundaries
    Theme_Keyword: Territorial boundaries

  Place:
    Place_Keyword_Thesaurus: None
    Place_Keyword: Mid-latitude
    Place_Keyword: Western Hemisphere
    Place_Keyword: Northern Hemisphere
    Place_Keyword: North America
    Place_Keyword: NAFTA
    Place_Keyword: North America Free Trade Agreement
    Place_Keyword: Canada
    Place_Keyword: Mexico
    Place_Keyword: United States

Access_Constraints: None
Use_Constraints:
  All data for Canada and other areas outside of Mexico and the United States of America are
  covered by the Geogratis License Agreement for Unrestricted Use of Digital Data; see
  http://geogratis.cgdi.gc.ca/geogratis/en/licence.jsp.

  No use constraints are applied to data for Mexico and the United States of America.
  Acknowledgment of Natural Resources Canada, Instituto Nacional de Estadística y Geografía,
  National Atlas of the United States, and (or) the Commission for Environmental Cooperation
  would be appreciated in products derived from these data.

Point_of_Contact:
  Contact_Information:
    Contact_Organization_Primary:
```

```
      Contact_Organization:
        Government of Canada, Natural Resources Canada, Mapping Information Branch, The
        Atlas of Canada
    Contact_Position: Atlas of Canada Client Services
    Contact_Address:
      Address_Type: Mailing address
      Address: Room 650-615 Booth Street
      City: Ottawa
      State_or_Province: Ontario
      Country: Canada
      Postal_Code: K1A 0E9
    Contact_Facsimile_Telephone: 1 613 947 2410
    Contact_Electronic_Mail_Address: info@atlas.gc.ca
Point_of_Contact:
  Contact_Information:
    Contact_Person_Primary:
      Contact_Person: Francisco Javier Jimenez Nava
      Contact_Organization: Dirección General de Geografía, INEGI
    Contact_Address:
      Address_Type: Mailing address
      Address: Av Heroe de Nacozari Sur 2301
      Address: Fracc Jardines del Parque
      City: Aguascalientes
      State_or_Province: Aguascalientes
      Country: Mexico
      Postal_Code: 20270
    Contact_Voice_Telephone: 52 449 910 5365
    Contact_Electronic_Mail_Address: francisco.jimenez@inegi.org.mx
Point_of_Contact:
  Contact_Information:
    Contact_Organization_Primary:
      Contact_Organization: National Atlas of the United States, U.S. Geological Survey
      Contact_Person: John Hutchinson
    Contact_Address:
      Address_Type: Mailing address
      Address: 47914 252nd Street
      City: Sioux Falls
      State_or_Province: South Dakota
      Country: USA
      Postal_Code: 57198-0001
    Contact_Voice_Telephone: 1 605 594 6049
    Contact_Facsimile_Telephone: 1 605 594 6529
    Contact_Electronic_Mail_Address: atlasmail@usgs.gov
Point_of_Contact:
  Contact_Information:
    Contact_Organization_Primary:
      Contact_Organization: Commission for Environmental Cooperation
    Contact_Address:
      Address_Type: Mailing address
      Address: 393, rue St-Jacques Ouest, Bureau 200
      City: Montréal
      State_or_Province: Québec
      Country: Canada
      Postal_Code: H2Y 1N9
    Contact_Voice_Telephone: 1 514 350 4300
    Contact_Facsimile_Telephone: 1 514 350 4314
    Contact_Electronic_Mail_Address: info@cec.org
```

Native_Data_Set_Environment:
Microsoft Windows XP Version 5.1 (Build 2600) Service Pack 3; ArcGIS Desktop 9.3.1 Service Pack 1 (Build 3500)

Data_Quality_Information:
Attribute_Accuracy:
Attribute_Accuracy_Report:
Where possible, attribute data were quality controlled by creating symbolized digital displays or hard copy plots, which were visually verified with digital or hard copy reference data. Attribute data that could not be verified in such a manner were interactively queried to check for anomalies such as duplication of data in fields requiring unique values and incorrect feature coding. Data for areas outside of Canada, Mexico, and the United States of America are only provided for context; Natural Resources Canada, Instituto Nacional de Estadística y Geografía, National Atlas of the United States, and the Commission for Environmental Cooperation assume no responsibility for the completeness, accuracy, or presentation of this data.
Logical_Consistency_Report:
Topology was built and a quality control of the political boundaries polygon data was done in order to ensure topological polygon structure. A quality control of the linear boundary file was done to ensure logical consistency with the corresponding polygon data.
Completeness_Report:
This data set includes political boundaries for all of North America, as shown at 1:10,000,000 scale. The data completeness reflects the content of the source data, the integration done by the cartographers, and the quality control of the content performed by each country.
Positional_Accuracy:
Horizontal_Positional_Accuracy:
Horizontal_Positional_Accuracy_Report:
Positional accuracy of the data is unknown. Positions are dependent on the accuracy of the source data and on the result of the generalization and integration processes. Data for areas outside of Canada, Mexico, and the United States of America are only provided for context; Natural Resources Canada, Instituto Nacional de Estadística y Geografía, National Atlas of the United States, and the Commission for Environmental Cooperation assume no responsibility for the completeness, accuracy, or presentation of this data.

State boundaries in Mexico are included for illustration purposes. They were obtained from diverse sources and INEGI does not guarantee and takes no responsibility for their legal validity and/or accuracy.

Lineage:
Source_Information:
Source_Citation:
Citation_Information:
Originator:
Government of Canada, Natural Resources Canada, Mapping Information Branch, The Atlas of Canada
Publication_Date: 2002
Title: 1:6,000,000 National Atlas Reference Map Series - Boundaries
Geospatial_Data_Presentation_Form: Vector digital data
Publication_Information:
Publication_Place: Ottawa, Ontario, Canada
Publisher:
Government of Canada, Natural Resources Canada, Mapping Information Branch, The Atlas of Canada
Source_Scale_Denominator: 6000000
Type_of_Source_Media: Online

```
  Source_Time_Period_of_Content:
    Time_Period_Information:
      Single_Date/Time:
        Calendar_Date: 2002
    Source_Currentness_Reference: Ground condition
  Source_Citation_Abbreviation: 6M CA Bdy
  Source_Contribution: Spatial and attribute information

Source_Information:
  Source_Citation:
    Citation_Information:
      Originator:
        Government of Canada, Natural Resources Canada, Mapping Information Branch, The
        Atlas of Canada
      Publication_Date: 2000
      Title: North America, National Atlas of Canada Reference Map Series
      Geospatial_Data_Presentation_Form: Vector digital data
      Publication_Information:
        Publication_Place: Ottawa, Ontario, Canada
        Publisher:
          Government of Canada, Natural Resources Canada, Mapping Information Branch, The
          Atlas of Canada
  Source_Scale_Denominator: 10000000
  Type_of_Source_Media: Online
  Source_Time_Period_of_Content:
    Time_Period_Information:
      Single_Date/Time:
        Calendar_Date: 2000
    Source_Currentness_Reference: Ground condition
  Source_Citation_Abbreviation: North America Reference
  Source_Contribution: Spatial information

Source_Information:
  Source_Citation:
    Citation_Information:
      Originator: Instituto Nacional de Estadística y Geografía
      Publication_Date: 2004
      Title: Conjunto de Datos Vectoriales Topográficos escala 1:4,000,000
      Geospatial_Data_Presentation_Form: Vector digital data
      Publication_Information:
        Publication_Place: Aguascalientes, Aguascalientes, Mexico
        Publisher:
          Instituto Nacional de Estadística y Geografía (INEGI)
  Source_Scale_Denominator: 4000000
  Type_of_Source_Media: CD-ROM
  Source_Time_Period_of_Content:
    Time_Period_Information:
      Range_of_Dates/Times:
        Beginning_Date: 19930101
        Ending_Date: 19970401
    Source_Currentness_Reference: Ground condition
  Source_Citation_Abbreviation: 4M Mexico
  Source_Contribution: Spatial and attribute information

Source_Information:
  Source_Citation:
    Citation_Information:
```

Originator:
United States Department of the Interior, U.S. Geological Survey, National Atlas of the United States
Publication_Date: Unpublished material
Title: 1:5,000,000 General Reference Map - Boundaries
Geospatial_Data_Presentation_Form: Vector digital data
Publication_Information:
Publication_Place: Reston, Virginia, USA
Publisher: U.S. Geological Survey, National Atlas of the United States
Source_Scale_Denominator: 5000000
Type_of_Source_Media: Internal file
Source_Time_Period_of_Content:
Time_Period_Information:
Single_Date/Time:
Calendar_Date: 1998
Source_Currentness_Reference: Date of data compilation
Source_Citation_Abbreviation: 5M USA Bdy
Source_Contribution: Spatial and attribute information

Source_Information:
Source_Citation:
Citation_Information:
Originator:
Government of Canada, Natural Resources Canada, Mapping Information Branch, The Atlas of Canada
Originator: Instituto Nacional de Estadística y Geografía
Originator:
United States Department of the Interior, U.S. Geological Survey, National Atlas of the United States
Publication_Date: 2004
Title: North American Atlas – Hydrography
Geospatial_Data_Presentation_Form: Vector digital data
Publication_Information:
Publication_Place: Ottawa, Ontario, Canada
Publisher:
Government of Canada, Natural Resources Canada, Mapping Information Branch, The Atlas of Canada
Publication_Information:
Publication_Place: Aguascalientes, Aguascalientes, Mexico
Publisher:
Instituto Nacional de Estadística y Geografía (INEGI)
Publication_Information:
Publication_Place: Reston, Virginia, USA
Publisher: U.S. Geological Survey, National Atlas of the United States
Publication_Information:
Publication_Place: Montréal, Québec, Canada
Publisher: Commission for Environmental Cooperation
Source_Scale_Denominator: 10000000
Type_of_Source_Media: Online
Source_Time_Period_of_Content:
Time_Period_Information:
Single_Date/Time:
Calendar_Date: 2004
Source_Currentness_Reference: Publication date
Source_Citation_Abbreviation: NAA Hydro
Source_Contribution: Spatial information

Source_Information:
Source_Citation:
Citation_Information:
Originator:
Government of Canada, Natural Resources Canada, Mapping Information Branch, The Atlas of Canada
Originator: Instituto Nacional de Estadística y Geografía
Originator:
United States Department of the Interior, U.S. Geological Survey, National Atlas of the United States
Publication_Date: 2004
Title: North American Atlas – Political Boundaries
Geospatial_Data_Presentation_Form: Vector digital data
Publication_Information:
Publication_Place: Ottawa, Ontario, Canada
Publisher:
Government of Canada, Natural Resources Canada, Mapping Information Branch, The Atlas of Canada
Publication_Information:
Publication_Place: Aguascalientes, Aguascalientes, Mexique
Publisher:
Instituto Nacional de Estadística y Geografía (INEGI)
Publication_Information:
Publication_Place: Reston, Virginia, USA
Publisher: U.S. Geological Survey, National Atlas of the United States
Publication_Information:
Publication_Place: Montréal, Québec, Canada
Publisher: Commission for Environmental Cooperation
Source_Scale_Denominator: 10000000
Type_of_Source_Media: Online
Source_Time_Period_of_Content:
Time_Period_Information:
Single_Date/Time:
Calendar_Date: 2004
Source_Currentness_Reference: Date of publication
Source_Citation_Abbreviation: NA_Bdy04
Source_Contribution: Spatial and attribute information

Source_Information:
Source_Citation:
Citation_Information:
Originator:
Government of Canada, Natural Resources Canada, Mapping Information Branch, The Atlas of Canada
Originator: Instituto Nacional de Estadística y Geografía
Originator:
United States Department of the Interior, U.S. Geological Survey, National Atlas of the United States
Publication_Date: 2006
Title: North American Atlas - Hydrography
Geospatial_Data_Presentation_Form: Vector digital data
Publication_Information:
Publication_Place: Ottawa, Ontario, Canada
Publisher:
Government of Canada, Natural Resources Canada, Mapping Information Branch, The Atlas of Canada
Publication_Information:

```
        Publication_Place: Aguascalientes, Aguascalientes, Mexique
        Publisher:
          Instituto Nacional de Estadística y Geografía (INEGI)
      Publication_Information:
        Publication_Place: Reston, Virginia, USA
        Publisher: U.S. Geological Survey, National Atlas of the United States
      Publication_Information:
        Publication_Place: Montréal, Québec, Canada
        Publisher: Commission for Environmental Cooperation
  Source_Scale_Denominator: 10000000
  Type_of_Source_Media: Online
  Source_Time_Period_of_Content:
    Time_Period_Information:
      Single_Date/Time:
        Calendar_Date: 2006
    Source_Currentness_Reference: Date of publication
  Source_Citation_Abbreviation: NA_Hydro06
  Source_Contribution: Spatial and attribute information

Source_Information:
  Source_Citation:
    Citation_Information:
      Originator:
        Government of Canada, Natural Resources Canada, Mapping Information Branch, The
        Atlas of Canada
      Originator: Instituto Nacional de Estadística y Geografía
      Originator:
        United States Department of the Interior, U.S. Geological Survey, National Atlas of
        the United States
      Publication_Date: 2006
      Title: North American Atlas - Political Boundaries
      Geospatial_Data_Presentation_Form: Vector digital data
      Publication_Information:
        Publication_Place: Ottawa, Ontario, Canada
        Publisher:
          Government of Canada, Natural Resources Canada, Mapping Information Branch, The
          Atlas of Canada
      Publication_Information:
        Publication_Place: Aguascalientes, Aguascalientes, Mexique
        Publisher:
          Instituto Nacional de Estadística y Geografía (INEGI)
      Publication_Information:
        Publication_Place: Reston, Virginia, USA
        Publisher: U.S. Geological Survey, National Atlas of the United States
      Publication_Information:
        Publication_Place: Montréal, Québec, Canada
        Publisher: Commission for Environmental Cooperation
  Source_Scale_Denominator: 10000000
  Type_of_Source_Media: Online
  Source_Time_Period_of_Content:
    Time_Period_Information:
      Single_Date/Time:
        Calendar_Date: 2006
    Source_Currentness_Reference: Date of publication
  Source_Citation_Abbreviation: NA_Bdy06
  Source_Contribution: Spatial and attribute information
```

```
Source_Information:
  Source_Citation:
    Citation_Information:
      Originator:
        Government of Canada, Natural Resources Canada, Mapping Information Branch, The
        Atlas of Canada
      Originator: Instituto Nacional de Estadística y Geografía
      Originator:
        United States Department of the Interior, U.S. Geological Survey, National Atlas of
        the United States
      Publication_Date: 2010
      Title: North American Atlas - Watersheds
      Geospatial_Data_Presentation_Form: Vector digital data
      Publication_Information:
        Publication_Place: Ottawa, Ontario, Canada
        Publisher:
          Government of Canada, Natural Resources Canada, Mapping Information Branch, The
          Atlas of Canada
      Publication_Information:
        Publication_Place: Aguascalientes, Aguascalientes, Mexique
        Publisher:
          Instituto Nacional de Estadística y Geografía (INEGI)
      Publication_Information:
        Publication_Place: Reston, Virginia, USA
        Publisher: U.S. Geological Survey, National Atlas of the United States
      Publication_Information:
        Publication_Place: Montréal, Québec, Canada
        Publisher: Commission for Environmental Cooperation
  Source_Scale_Denominator: 1000000
  Type_of_Source_Media: Online
  Source_Time_Period_of_Content:
    Time_Period_Information:
      Single_Date/Time:
        Calendar_Date: 2010
    Source_Currentness_Reference: Date of publication
  Source_Citation_Abbreviation: NA_Watersheds
  Source_Contribution: Spatial and attribute information

Source_Information:
  Source_Citation:
    Citation_Information:
      Originator: U.S. Geological Survey
      Publication_Date: Unknown
      Title: 1:24,000 Digital Raster Graphics
      Geospatial_Data_Presentation_Form: Vector digital data
      Publication_Information:
        Publication_Place: Reston, Virginia, USA
        Publisher: U.S. Geological Survey
  Source_Scale_Denominator: 24000
  Type_of_Source_Media: Online
  Source_Time_Period_of_Content:
    Time_Period_Information:
      Single_Date/Time:
        Calendar_Date: Unknown
    Source_Currentness_Reference: Publication date
  Source_Citation_Abbreviation: 24K DRGs
  Source_Contribution: Spatial information
```

Source_Information:
  Source_Citation:
    Citation_Information:
      Originator:
        Government of Canada, Natural Resources Canada, Mapping Information Branch, The
        Atlas of Canada
      Publication_Date: Unpublished material
      Title: Updates to North American Atlas - Hydrography
      Geospatial_Data_Presentation_Form: Vector digital data
  Source_Scale_Denominator: 10000000
  Type_of_Source_Media: Internal file
  Source_Time_Period_of_Content:
    Time_Period_Information:
      Single_Date/Time:
        Calendar_Date: 2008
    Source_Currentness_Reference: Date of initial edit
  Source_Citation_Abbreviation: NA_Hydro_updates
  Source_Contribution: Spatial and attribute information

Source_Information:
  Source_Citation:
    Citation_Information:
      Originator:
        Government of Canada, Natural Resources Canada, Canada Centre for Remote
        Sensing, The Atlas of Canada
      Publication_Date: 2008
      Title: 1:1,000,000 Political Boundaries
      Geospatial_Data_Presentation_Form: Vector digital data
      Publication_Information:
        Publication_Place: Ottawa, Ontario, Canada
        Publisher: Government of Canada
  Source_Scale_Denominator: 1000000
  Type_of_Source_Media: Internal file
  Source_Time_Period_of_Content:
    Time_Period_Information:
      Single_Date/Time:
        Calendar_Date: 20080917
    Source_Currentness_Reference: Download date
  Source_Citation_Abbreviation: BNDLAM
  Source_Contribution: Spatial and attribute information

Source_Information:
  Source_Citation:
    Citation_Information:
      Originator:
        Government of Canada, Natural Resources Canada, Mapping Information Branch, The
        Atlas of Canada
      Originator: Instituto Nacional de Estadística y Geografía
      Originator:
        United States Department of the Interior, U.S. Geological Survey, National Atlas of
        the United States
      Publication_Date: 2010
      Title: North American Atlas - Hydrography
      Geospatial_Data_Presentation_Form: Vector digital data
      Publication_Information:
        Publication_Place: Ottawa, Ontario, Canada

Publisher:
Government of Canada, Natural Resources Canada, Mapping Information Branch, The Atlas of Canada
Publication_Information:
Publication_Place: Aguascalientes, Aguascalientes, Mexique
Publisher:
Instituto Nacional de Estadística y Geografía (INEGI)
Publication_Information:
Publication_Place: Reston, Virginia, USA
Publisher: U.S. Geological Survey, National Atlas of the United States
Publication_Information:
Publication_Place: Montréal, Québec, Canada
Publisher: Commission for Environmental Cooperation
Source_Scale_Denominator: 10000000
Type_of_Source_Media: Online
Source_Time_Period_of_Content:
Time_Period_Information:
Single_Date/Time:
Calendar_Date: 2010
Source_Currentness_Reference: Date of publication
Source_Citation_Abbreviation: NA_Hydro10
Source_Contribution: Spatial and attribute information

Process_Step:
Process_Description:
Linework from the 1:6,000,000 Atlas of Canada boundary file was fitted to the North American Atlas - Hydrography layer. The attribute for the country was added to the data.
Source_Used_Citation_Abbreviation: 6M CA Bdy
Source_Used_Citation_Abbreviation: NAA Hydro
Process_Date: 2003

Process_Step:
Process_Description:
Linework from the 1:5,000,000 United States General Reference Map was fitted to the North American Atlas - Hydrography layer. The attribute for the country was added to the data.
Source_Used_Citation_Abbreviation: 5M USA Bdy
Source_Used_Citation_Abbreviation: NAA Hydro
Process_Date: 2003

Process_Step:
Process_Description:
Linework from the 1:4,000,000 Topographical Dataset for Mexico was fitted to the North American Atlas - Hydrography layer. The attribute for the country was added to the data.
Source_Used_Citation_Abbreviation: 4M Mexico
Source_Used_Citation_Abbreviation: NAA Hydro
Process_Date: 2003

Process_Step:
Process_Description:
Boundaries for regions outside Canada, Mexico, and the United States of America were compiled from the 1:10,000,000 North America, National Atlas of Canada Reference Map Series data set. Linework was fitted to the North American Atlas - Hydrography layer. The attribute for foreign country was added to the data.
Source_Used_Citation_Abbreviation: North America Reference

Source_Used_Citation_Abbreviation: NAA Hydro
Process_Date: 2004

Process_Step:
Process_Description:
Linework was cartographically adjusted along country boundaries to ensure correct connection of adjacent boundaries and attributes were added.
Process_Date: 2004

Process_Step:
Process_Description:
Because the linework came from four different sources and scales, a final cartographic review was done to the full boundary file to ensure cartographic compatibility at 1:10,000,000 scale. Each country then performed a final cartographic check to verify the selection and position of the linework. The attributes were also verified.
Process_Date: 2004
Source_Produced_Citation_Abbreviation: NA_Bdy04

Process_Step:
Process_Description:
Boundary lines that followed hydrography features (rivers and lakes) in the United States were adjusted to fit the new hydrography positions. Attributes and geometry were checked to allow for corrections before release.
Source_Used_Citation_Abbreviation: NA_Bdy04
Source_Used_Citation_Abbreviation: NA_Hydro06
Process_Date: 2006
Source_Produced_Citation_Abbreviation: NA_Bdy06

Process_Step:
Process_Description:
The boundaries data were added to a geodatabase that included North American watersheds and other North American Atlas base layers in a Lambert Azimuthal Equal Area projection.

In the process of creating the 2010 North American Atlas - Watersheds data set, a number of discrepancies between the watershed boundaries and the existing North American Atlas base layers were identified. Resulting adjustments made to the hydrography layer necessitated changes to the international boundary in the political boundaries layer. A visual comparison of the boundaries and watersheds revealed three places (East Neebish Channel, Mary's River by Little Lake, and Niagara River from Buffalo NY up to Lake Ontario) where a political boundary crossed the updated shoreline for the Great Lakes. The political boundaries were adjusted in these areas to the center of the watershed unit (the lake); the 24K DRGs were used as reference for placement of the adjusted boundary. In addition, the international boundary was adjusted between longitudes 70° 17'7" W and 71° 24' 12". The international boundary in this area is aligned with the watershed boundary; the boundary line was adjusted to match the updated watershed boundary. The boundary polygons were updated based on the boundary line edits.
Source_Used_Citation_Abbreviation: NA_Bdy06
Source_Used_Citation_Abbreviation: NA Hydro_updates
Source_Used_Citation_Abbreviation: NA_watersheds
Source_Used_Citation_Abbreviation: 24K DRG
Process_Date: 200810

Process_Step:
Process_Description:
The political boundaries required additional editing because some provincial boundaries

follow rivers or are defined as height-of-land, and many of these coincide with North American Watershed boundaries, but the existing political boundaries were of lower absolute locational accuracy than the new watersheds. The political boundaries were upgraded.

The 1:1-million political boundaries coverage BNDLAM was copied from an internal Atlas of Canada repository. Segments of internal (provincial/territorial) boundaries that were coincident with streams or watershed boundaries were identified and generalized using the ArcGIS Smooth Line tool using the PAEK option with a tolerance of 6000 m, followed by some minor manual modification. (Smoothed sections were YK/NT, BC/AB, QC/NL, ON/QC along the Ottawa River, and QC/NB along the Restigouche River.)

Because most of the remaining political boundaries were relatively simple straight-line segments, it was decided to replace the entire set of Canadian provincial/territorial boundaries. These boundary segments were generalized or densified to achieve a vertex density of about 3000-5000 metres. All of the Canadian political boundary lines were deleted from the North American Atlas – Political Boundaries file and replaced with the newly updated boundaries from the process above. The locational accuracy of the internal Canadian boundaries is now much improved, and they shouldn’t need to be revisited in the future.

Source_Used_Citation_Abbreviation: BNDLAM
Source_Used_Citation_Abbreviation: NA_Hydro_updates
Source_Used_Citation_Abbreviation: NA_watersheds
Process_Date: 200908

Process_Step:
Process_Description:
The boundaries were checked against the updated hydrography for mismatches along coastlines. Errors were corrected so that the two files are in agreement.
Source_Used_Citation_Abbreviation: NA_Hydro10
Process_Date: 201005

Process_Step:
Process_Description:
Corrections were made to several attribute values to fix errors identified in the quality-control process.

The data set was saved in an unprojected format for distribution by Atlas of Canada, Instituto Nacional de Estadística y Geografía, and National Atlas of the United States. This Political Boundaries data and all other base data sets in the North American Environmental Atlas are maintained by the Atlas as a geodatabase in a Lambert Azimuthal Equal Area projection.
Process_Date: 201007

Spatial_Data_Organization_Information:
Direct_Spatial_Reference_Method: Vector
Point_and_Vector_Object_Information:
SDTS_Terms_Description:
SDTS_Point_and_Vector_Object_Type: Complete chain
Point_and_Vector_Object_Count: 3069
SDTS_Terms_Description:
SDTS_Point_and_Vector_Object_Type: GT-polygon composed of chains
Point_and_Vector_Object_Count: 1833

Spatial_Reference_Information:
Horizontal_Coordinate_System_Definition:
Geographic:

```
      Latitude_Resolution: 0.000001
      Longitude_Resolution: 0.000001
      Geographic_Coordinate_Units: Decimal degrees
    Geodetic_Model:
      Horizontal_Datum_Name: North American Datum of 1983
      Ellipsoid_Name: Geodetic Reference System 80
      Semi-major_Axis: 6378137.000000
      Denominator_of_Flattening_Ratio: 298.257222

Entity_and_Attribute_Information:
  Detailed_Description:
    Entity_Type:
      Entity_Type_Label: Boundary line
      Entity_Type_Definition:
        The limiting line of jurisdictional authority for various levels of government, or the
        International Date Line.
      Entity_Type_Definition_Source: North American Atlas GIS Group
    Attribute:
      Attribute_Label: Shape
      Attribute_Definition: The representation of the entity in the data.
      Attribute_Definition_Source: Commission for Environmental Cooperation
      Attribute_Domain_Values:
        Enumerated_Domain:
          Enumerated_Domain_Value: PolyLine
          Enumerated_Domain_Value_Definition:
            A 1-dimensional element that may or may not surround a 2-dimensional element.
          Enumerated_Domain_Value_Definition_Source: ESRI GIS software
    Attribute:
      Attribute_Label: Uident
      Attribute_Definition:
        The unique identifier.  A unique number that is assigned to each feature in the North
        American Atlas data.  The last two digits of the identifier are a suffix indicating the
        map layer.
      Attribute_Definition_Source: North American Atlas GIS Group
      Attribute_Domain_Values:
        Range_Domain:
          Range_Domain_Minimum: 32103
          Range_Domain_Maximum: 328503
    Attribute:
      Attribute_Label: Type
      Attribute_Definition: The type of boundary line
      Attribute_Definition_Source: North American Atlas GIS Group
      Attribute_Domain_Values:
        Enumerated_Domain:
          Enumerated_Domain_Value: 10
          Enumerated_Domain_Value_Definition:
            The line represents the boundary between Mexico and the United
            States of America.
          Enumerated_Domain_Value_Definition_Source:
            North American Atlas GIS Group
      Attribute_Domain_Values:
        Enumerated_Domain:
          Enumerated_Domain_Value: 11
          Enumerated_Domain_Value_Definition:
            The line represents the boundary between Mexico and Guatemala, or
            between Mexico and Belize.
          Enumerated_Domain_Value_Definition_Source:
```

```
      North American Atlas GIS Group
Attribute_Domain_Values:
  Enumerated_Domain:
    Enumerated_Domain_Value: 12
    Enumerated_Domain_Value_Definition:
      The line represents the boundary between Canada and the United
      States of America.
    Enumerated_Domain_Value_Definition_Source:
      North American Atlas GIS Group
Attribute_Domain_Values:
  Enumerated_Domain:
    Enumerated_Domain_Value: 15
    Enumerated_Domain_Value_Definition:
      The line represents the coastline of the ocean.
    Enumerated_Domain_Value_Definition_Source:
      North American Atlas GIS Group
Attribute_Domain_Values:
  Enumerated_Domain:
    Enumerated_Domain_Value: 16
    Enumerated_Domain_Value_Definition:
      The line represents the shoreline of the Great Lakes
    Enumerated_Domain_Value_Definition_Source:
      North American Atlas GIS Group
Attribute_Domain_Values:
  Enumerated_Domain:
    Enumerated_Domain_Value: 35
    Enumerated_Domain_Value_Definition:
      The line represents an international boundary between non-NAFTA
      countries.
    Enumerated_Domain_Value_Definition_Source:
      North American Atlas GIS Group
Attribute_Domain_Values:
  Enumerated_Domain:
    Enumerated_Domain_Value: 36
    Enumerated_Domain_Value_Definition:
      The line represents a Provincial, State, or Territorial boundary.
    Enumerated_Domain_Value_Definition_Source:
      North American Atlas GIS Group
Attribute_Domain_Values:
  Enumerated_Domain:
    Enumerated_Domain_Value: 39
    Enumerated_Domain_Value_Definition:
      The line represents the boundary between Canada and Greenland.
    Enumerated_Domain_Value_Definition_Source:
      North American Atlas GIS Group
Attribute_Domain_Values:
  Enumerated_Domain:
    Enumerated_Domain_Value: 42
    Enumerated_Domain_Value_Definition:
      The line represents the International Date Line.
    Enumerated_Domain_Value_Definition_Source:
      North American Atlas GIS Group
Attribute_Domain_Values:
  Enumerated_Domain:
    Enumerated_Domain_Value: 89
    Enumerated_Domain_Value_Definition:
      The line represents the hemisphere dividing line.
```

```
      Enumerated_Domain_Value_Definition_Source:
        North American Atlas GIS Group
  Attribute_Domain_Values:
    Enumerated_Domain:
      Enumerated_Domain_Value: 99
      Enumerated_Domain_Value_Definition:
        The line represents the outer limit of the data set.
      Enumerated_Domain_Value_Definition_Source:
        North American Atlas GIS Group
Attribute:
  Attribute_Label: Country
  Attribute_Definition:
    The country in which the jurisdictional boundary line exists.
  Attribute_Definition_Source: North American Atlas GIS Group
  Attribute_Domain_Values:
    Enumerated_Domain:
      Enumerated_Domain_Value: CAN
      Enumerated_Domain_Value_Definition: The boundary line is in Canada.
      Enumerated_Domain_Value_Definition_Source:
        North American Atlas GIS Group
  Attribute_Domain_Values:
    Enumerated_Domain:
      Enumerated_Domain_Value: CAN USA
      Enumerated_Domain_Value_Definition:
        The feature coincides with a political boundary.
      Enumerated_Domain_Value_Definition_Source:
        North American Atlas GIS Group
  Attribute_Domain_Values:
    Enumerated_Domain:
      Enumerated_Domain_Value: FN
      Enumerated_Domain_Value_Definition:
        The boundary line is outside of Canada, Mexico, and the United
        States of America.
      Enumerated_Domain_Value_Definition_Source:
        North American Atlas GIS Group
  Attribute_Domain_Values:
    Enumerated_Domain:
      Enumerated_Domain_Value: MEX
      Enumerated_Domain_Value_Definition: The boundary line is in Mexico.
      Enumerated_Domain_Value_Definition_Source:
        North American Atlas GIS Group
  Attribute_Domain_Values:
    Enumerated_Domain:
      Enumerated_Domain_Value: MEX FN
      Enumerated_Domain_Value_Definition:
        The feature coincides with a political boundary.
      Enumerated_Domain_Value_Definition_Source:
        North American Atlas GIS Group
  Attribute_Domain_Values:
    Enumerated_Domain:
      Enumerated_Domain_Value: MEX USA
      Enumerated_Domain_Value_Definition:
        The feature coincides with a political boundary.
      Enumerated_Domain_Value_Definition_Source:
```

North American Atlas GIS Group
Attribute_Domain_Values:
Enumerated_Domain:
Enumerated_Domain_Value: NONE
Enumerated_Domain_Value_Definition:
The line represents the limit of the data set or the hemisphere dividing line and no country is specified.
Enumerated_Domain_Value_Definition_Source:
North American Atlas GIS Group
Attribute_Domain_Values:
Enumerated_Domain:
Enumerated_Domain_Value: USA
Enumerated_Domain_Value_Definition:
The boundary line is in the United States of America.
Enumerated_Domain_Value_Definition_Source:
North American Atlas GIS Group
Attribute:
Attribute_Label: Edit_date
Attribute_Definition: The date on which edits were made to the element.
Attribute_Definition_Source: North American Atlas GIS Group
Attribute_Domain_Values:
Enumerated_Domain:
Enumerated_Domain_Value: 20060803
Enumerated_Domain_Value_Definition: Edits were made on August 3, 2006.
Enumerated_Domain_Value_Definition_Source:
North American Atlas GIS Group
Attribute_Domain_Values:
Enumerated_Domain:
Enumerated_Domain_Value: 20090827
Enumerated_Domain_Value_Definition: Edits were made on August 27, 2009.
Enumerated_Domain_Value_Definition_Source:
North American Atlas GIS Group
Attribute_Domain_Values:
Enumerated_Domain:
Enumerated_Domain_Value: 20100505
Enumerated_Domain_Value_Definition: Edits were made on May 5, 2010.
Enumerated_Domain_Value_Definition_Source:
North American Atlas GIS Group
Attribute_Domain_Values:
Enumerated_Domain:
Enumerated_Domain_Value: 20100510
Enumerated_Domain_Value_Definition: Edits were made on May 10, 2010.
Enumerated_Domain_Value_Definition_Source:
North American Atlas GIS Group
Attribute_Domain_Values:
Enumerated_Domain:
Enumerated_Domain_Value: 20100511
Enumerated_Domain_Value_Definition: Edits were made on May 11, 2010.
Enumerated_Domain_Value_Definition_Source:
North American Atlas GIS Group
Attribute:
Attribute_Label: Edit
Attribute_Definition: The type of edit performed on the element.
Attribute_Definition_Source: North American Atlas GIS Group
Attribute_Domain_Values:
Enumerated_Domain:

```
        Enumerated_Domain_Value: ADD
        Enumerated_Domain_Value_Definition:
          The line was not in the earliest version of the data set, but was added on the date
          listed.
        Enumerated_Domain_Value_Definition_Source:
          North American Atlas GIS Group
    Attribute_Domain_Values:
      Enumerated_Domain:
        Enumerated_Domain_Value: EDT
        Enumerated_Domain_Value_Definition:
          The line has been changed from its previous shape, position, or attribution.
        Enumerated_Domain_Value_Definition_Source:
          North American Atlas GIS Group
    Attribute_Domain_Values:
      Enumerated_Domain:
        Enumerated_Domain_Value: NEW
        Enumerated_Domain_Value_Definition:
          The line existed in the data set when the data set was new.
        Enumerated_Domain_Value_Definition_Source:
          North American Atlas GIS Group
  Attribute:
    Attribute_Label: Shape_leng
    Attribute_Definition: The length of the line.  Units are unknown.
    Attribute_Definition_Source: North American Atlas GIS Group
    Attribute_Domain_Values:
      Range_Domain:
        Range_Domain_Minimum: 59.14720325010
        Range_Domain_Maximum: 4980000.00002999980

Detailed_Description:
  Entity_Type:
    Entity_Type_Label: Jurisdictional area
    Entity_Type_Definition:
      An area for which one level of government has jurisdictional authority.
    Entity_Type_Definition_Source: North American Atlas GIS Group
  Attribute:
    Attribute_Label: Shape
    Attribute_Definition: The representation of the entity in the data.
    Attribute_Definition_Source: Commission for Environmental Cooperation
    Attribute_Domain_Values:
      Enumerated_Domain:
        Enumerated_Domain_Value: Polygon
        Enumerated_Domain_Value_Definition: A 2-dimensional element
        Enumerated_Domain_Value_Definition_Source: ESRI GIS software
  Attribute:
    Attribute_Label: Uident
    Attribute_Definition:
      The unique identifier.  A unique number that is assigned to each feature
      in the North American Atlas data.  The last two digits of the identifier
      are a suffix indicating the map layer.
    Attribute_Definition_Source: North American Atlas GIS Group
    Attribute_Domain_Values:
      Range_Domain:
        Range_Domain_Minimum: 104
        Range_Domain_Maximum: 184404
  Attribute:
    Attribute_Label: Name
```

```
    Attribute_Definition:
      The official name of the area.  Canadian names may be listed in both English and
      French.  Names of foreign areas may be listed in English, French, and (or) Spanish.
    Attribute_Definition_Source: North American Atlas GIS Group
    Attribute_Domain_Values:
      Codeset_Domain:
        Codeset_Name: The Canadian Geographical Names Data Base
        Codeset_Source:
          The Canadian Permanent Committee on Geographical Names
    Attribute_Domain_Values:
      Codeset_Domain:
        Codeset_Name: Toponymic data set 1:4,000,000
        Codeset_Source:
          Instituto Nacional de Estadística y Geografía(INEGI)
    Attribute_Domain_Values:
      Codeset_Domain:
        Codeset_Name: Geographic Names Information System
        Codeset_Source: U.S. Board on Geographic Names
    Attribute_Domain_Values:
      Enumerated_Domain:
        Enumerated_Domain_Value: water/agua/d'eau
        Enumerated_Domain_Value_Definition:
          The area represents the ocean or one of the Great Lakes and no name is assigned.
        Enumerated_Domain_Value_Definition_Source:
          North American Atlas GIS Group
  Attribute:
    Attribute_Label: Country
    Attribute_Definition: The country in which the jurisdictional area exists.
    Attribute_Definition_Source: North American Atlas GIS Group
    Attribute_Domain_Values:
      Enumerated_Domain:
        Enumerated_Domain_Value: CAN
        Enumerated_Domain_Value_Definition: The area is in Canada.
        Enumerated_Domain_Value_Definition_Source:
          North American Atlas GIS Group
    Attribute_Domain_Values:
      Enumerated_Domain:
        Enumerated_Domain_Value: MEX
        Enumerated_Domain_Value_Definition: The area is in Mexico.
        Enumerated_Domain_Value_Definition_Source:
          North American Atlas GIS Group
    Attribute_Domain_Values:
      Enumerated_Domain:
        Enumerated_Domain_Value: USA
        Enumerated_Domain_Value_Definition:
          The area is in the United States of America.
        Enumerated_Domain_Value_Definition_Source:
          North American Atlas GIS Group
    Attribute_Domain_Values:
      Enumerated_Domain:
        Enumerated_Domain_Value: FN
        Enumerated_Domain_Value_Definition:
          The area is outside of Canada, Mexico, and the United States of
          America.
        Enumerated_Domain_Value_Definition_Source:
          North American Atlas GIS Group
```

```
  Attribute_Domain_Values:
    Enumerated_Domain:
      Enumerated_Domain_Value: water/agua/d’eau
      Enumerated_Domain_Value_Definition:
        The area represents the ocean and no country is specified.
      Enumerated_Domain_Value_Definition_Source:
        North American Atlas GIS Group
Attribute:
  Attribute_Label: StateAbb
  Attribute_Definition:
    The International Organization for Standards (ISO) code for the State,
    Province,or Territory.  The code consists of a two-character country code
    followed by a two- or three-character State, Province, or Territory code.
  Attribute_Definition_Source: North American Atlas GIS Group
  Attribute_Domain_Values:
    Codeset_Domain:
      Codeset_Name: ISO 3166-2 codes for Canada
      Codeset_Source:
        http://www.commondatahub.com/live/geography/state_province_region/iso_3166_2_state_code
  Attribute_Domain_Values:
    Codeset_Domain:
      Codeset_Name: ISO 3166-2 codes for Mexico
      Codeset_Source:
        http://www.commondatahub.com/live/geography/state_province_region/iso_3166_2_state_code
  Attribute_Domain_Values:
    Codeset_Domain:
      Codeset_Name: ISO 3166-2 codes for the United States
      Codeset_Source:
        http://www.commondatahub.com/live/geography/state_province_region/iso_3166_2_state_code
  Attribute_Domain_Values:
    Enumerated_Domain:
      Enumerated_Domain_Value: <blank>
      Enumerated_Domain_Value_Definition:
        The area represents the ocean; the Great Lakes; an area outside of Canada, Mexico,
        or the United States of America; or another area where the state, province, or
        territory is not specified.
      Enumerated_Domain_Value_Definition_Source:
        North American Atlas GIS Group
Attribute:
  Attribute_Label: Edit_date
  Attribute_Definition: The date on which edits were made to the element.
  Attribute_Definition_Source: North American Atlas GIS Group
  Attribute_Domain_Values:
    Enumerated_Domain:
      Enumerated_Domain_Value: 20060803
      Enumerated_Domain_Value_Definition: Edits were made on August 3, 2006.
      Enumerated_Domain_Value_Definition_Source:
        North American Atlas GIS Group
  Attribute_Domain_Values:
    Enumerated_Domain:
      Enumerated_Domain_Value: 20090827
      Enumerated_Domain_Value_Definition: Edits were made on August 27, 2009.
      Enumerated_Domain_Value_Definition_Source:
        North American Atlas GIS Group
  Attribute_Domain_Values:
    Enumerated_Domain:
```

```
        Enumerated_Domain_Value: 20100505
        Enumerated_Domain_Value_Definition: Edits were made on May 5, 2010.
        Enumerated_Domain_Value_Definition_Source:
          North American Atlas GIS Group
    Attribute_Domain_Values:
      Enumerated_Domain:
        Enumerated_Domain_Value: 20100510
        Enumerated_Domain_Value_Definition: Edits were made on May 10, 2010.
        Enumerated_Domain_Value_Definition_Source:
          North American Atlas GIS Group
    Attribute_Domain_Values:
      Enumerated_Domain:
        Enumerated_Domain_Value: 20100511
        Enumerated_Domain_Value_Definition: Edits were made on May 11, 2010.
        Enumerated_Domain_Value_Definition_Source:
          North American Atlas GIS Group
  Attribute:
    Attribute_Label: Edit
    Attribute_Definition: The type of edit performed on the element.
    Attribute_Definition_Source: North American Atlas GIS Group
    Attribute_Domain_Values:
      Enumerated_Domain:
        Enumerated_Domain_Value: EDT
        Enumerated_Domain_Value_Definition:
          The area has been changed from its previous shape, position, or attribution.
        Enumerated_Domain_Value_Definition_Source:
          North American Atlas GIS Group
    Attribute_Domain_Values:
      Enumerated_Domain:
        Enumerated_Domain_Value: NEW
        Enumerated_Domain_Value_Definition:
          The area existed in the data set when the data set was new.
        Enumerated_Domain_Value_Definition_Source:
          North American Atlas GIS Group
  Attribute:
    Attribute_Label: Shape_leng
    Attribute_Definition: The length of the perimeter line.  Units are unknown.
    Attribute_Definition_Source: North American Atlas GIS Group
    Attribute_Domain_Values:
      Range_Domain:
        Range_Domain_Minimum: 3311.5554338000
        Range_Domain_Maximum: 220301964.903000000
  Attribute:
    Attribute_Label: Shape_area
    Attribute_Definition: The size of the polygon.  Units are unknown.
    Attribute_Definition_Source: North American Atlas GIS Group
    Attribute_Domain_Values:
      Range_Domain:
        Range_Domain_Minimum: 814697.89640099998
        Range_Domain_Maximum: 33509088749800.0000

Distribution_Information:
  Distributor:
    Contact_Information:
      Contact_Organization_Primary:
        Contact_Organization:
          Government of Canada, Natural Resources Canada, Earth Sciences Sector, Data
```

```
        Management and Dissemination Branch
      Contact_Address:
        Address_Type: Mailing address
        Address: Room 650-615 Booth Street
        City: Ottawa
        State_or_Province: Ontario
        Country: Canada
        Postal_Code: K1A 0E9
      Contact_Facsimile_Telephone: 1 613 947 2410
      Contact_Electronic_Mail_Address: info@GeoGratis.gc.ca
Distribution_Information:
  Distributor:
    Contact_Information:
      Contact_Organization_Primary:
        Contact_Organization: INEGI
        Contact_Person: Jose Luis Ornelas de Anda
      Contact_Address:
        Address_Type: Mailing address
        Address: Avenida Héroe de Nacozari Sur 2301
        Address: Fraccionamiento Jardines del Parque
        City: Aguascalientes
        State_or_Province: Aguascalientes
        Country: Mexico
        Postal_Code: 20270
      Contact_Voice_Telephone: 52 449 910 5300 ext 5856
      Contact_Electronic_Mail_Address: jose.ornelas@inegi.org.mx
Distribution_Information:
  Distributor:
    Contact_Information:
      Contact_Organization_Primary:
        Contact_Organization: U.S. Geological Survey
      Contact_Address:
        Address_Type: Mailing address
        Address: 47914 252nd Street
        City: Sioux Falls
        State_or_Province: South Dakota
        Country: USA
        Postal_Code: 57198-0001
      Contact_Voice_Telephone: 1 800 252 4547
      Contact_Voice_Telephone: 1 605 594 6151
      Contact_TDD/TTY_Telephone: 1 605 594 6933
      Contact_Facsimile_Telephone: 1 605 594 6589
      Contact_Electronic_Mail_Address: custserv@usgs.gov
Distribution_Information:
  Distributor:
    Contact_Information:
      Contact_Organization_Primary:
        Contact_Organization:
          Commission for Environmental Cooperation
      Contact_Address:
        Address_Type: Mailing address
        Address: 393, rue St-Jacques Ouest, Bureau 200
        City: Montréal
        State_or_Province: Québec
        Country: Canada
        Postal_Code: H2Y 1N9
      Contact_Voice_Telephone: 1 514 350 4300
```

```
      Contact_Facsimile_Telephone: 1 514 350 4314
      Contact_Electronic_Mail_Address: info@cec.org

  Distribution_Liability:
    Although these data have been processed successfully on computer systems at Natural
    Resources Canada, Instituto Nacional de Estadística y Geografía, National Atlas of the
    United States, and the Commission for Environmental Cooperation, no warranty expressed or
    implied is made by these agencies regarding the utility of the data on any other system,
    nor shall the act of distribution constitute any such warranty.  No responsibility is
    assumed by these agencies in the use of these data.
  Standard_Order_Process:
    Digital_Form:
      Digital_Transfer_Information:
        Format_Name: ESRI Shapefile
      Digital_Transfer_Option:
        Online_Option:
          Computer_Contact_Information:
            Network_Address:
              Network_Resource_Name:
                http://geogratis.gc.ca/download/frameworkdata/North_America_Atlas10M/boundaries/
              Network_Resource_Name: http://antares.inegi.org.mx/atlas_norteamerica/
              Network_Resource_Name: http://nationalatlas.gov/atlasftp-na.html
              Network_Resource_Name: http://www.cec.org/naatlas/
    Digital_Form:
      Digital_Transfer_Information:
        Format_Name: MXD
      Digital_Transfer_Option:
        Online_Option:
          Computer_Contact_Information:
            Network_Address:
              Network_Resource_Name: http://www.cec.org/naatlas
    Digital_Form:
      Digital_Transfer_Information:
        Format_Name: Layer package
      Digital_Transfer_Option:
        Online_Option:
          Computer_Contact_Information:
            Network_Address:
              Network_Resource_Name: http://www.cec.org/naatlas
    Digital_Form:
      Digital_Transfer_Information:
        Format_Name: GeoPDF
      Digital_Transfer_Option:
        Online_Option:
          Computer_Contact_Information:
            Network_Address:
              Network_Resource_Name: http://www.cec.org/naatlas

    Fees:
      Gratuit-Free

Metadata_Reference_Information:
  Metadata_Date: 20111108
  Metadata_Contact:
    Contact_Information:
      Contact_Organization_Primary:
        Contact_Organization:
```

```
      Government of Canada, Natural Resources Canada, Mapping Information Branch, The
      Atlas of Canada
    Contact_Position: Atlas of Canada Client Services
    Contact_Address:
      Address_Type: Mailing address
      Address: Room 650-615 Booth Street
      City: Ottawa
      State_or_Province: Ontario
      Country: Canada
      Postal_Code: K1A 0E9
    Contact_Facsimile_Telephone: 1 613 947 2410
    Contact_Electronic_Mail_Address: info@atlas.gc.ca

Metadata_Contact:
 Contact_Information:
    Contact_Organization_Primary:
      Contact_Organization: Dirección General de Geografía, INEGI
    Contact_Position: Administrador de Metadatos Geográficos
    Contact_Address:
      Address_Type: Mailing address
      Address: Av Heroe de Nacozari Sur 2301
      Address: Fracc Jardines del Parque
      City: Aguascalientes
      State_or_Province: Aguascalientes
      Country: Mexico
      Postal_Code: 20270
    Contact_Voice_Telephone: 52 449 910 53 00 ext 5856
    Contact_Electronic_Mail_Address: rafael.arrioja@inegi.org.mx

Metadata_Contact:
  Contact_Information:
    Contact_Person_Primary:
      Contact_Person: Terry Higgins
      Contact_Organization: National Atlas of the United States
    Contact_Address:
      Address_Type: Mailing address
      Address: 1400 Independence Rd.
      Address: MS-700
      City: Rolla
      State_or_Province: MO
      Postal_Code: 65401-2602
      Country: USA
    Contact_Voice_Telephone: 1 573 308 3592
    Contact_Electronic_Mail_Address: atlasmail@usgs.gov
Metadata_Contact:
  Contact_Information:
    Contact_Organization_Primary:
      Contact_Organization:
        Commission for Environmental Cooperation
    Contact_Address:
      Address_Type: Mailing address
      Address: 393, rue St-Jacques Ouest, Bureau 200
      City: Montréal
      State_or_Province: Québec
      Country: Canada
      Postal_Code: H2Y 1N9
    Contact_Voice_Telephone: 1 514 350 4300
```

```
    Contact_Facsimile_Telephone: 1 514 350 4314
    Contact_Electronic_Mail_Address: info@cec.org
Metadata_Standard_Name:
  FGDC Content Standards for Digital Geospatial Metadata
Metadata_Standard_Version: FGDC-STD-001-1998
```
