# Supplementary material for: Deep learning workflow to support in-flight processing of digital aerial imagery for wildlife population surveys
Source: PLoS One. 2024 Apr 3;19(4):e0288121. doi: 10.1371/journal.pone.0288121 (PMC10990224; doi:10.1371/journal.pone.0288121)
Supplement: Attachment — Submitted filename: Response to Revieweres.pdf [file pone.0288121.s004.pdf]

Dear editors and reviewers:

We appreciate your thoughtful feedback on our manuscript “Deep learning workflow to support in-flight processing of digital aerial imagery for wildlife population surveys”. Reviewers consider our work as interesting and effective for automating wildlife population surveys. We next address each reviewer’s concerns respectively.

[R1. Lack of novelty]
We clarify that this paper does not aim at proposing new classification / detection algorithms, but an automated workflow to support large-scale wildlife surveys. Endowed by the great success of deep learning algorithms, we alleviate the painful need of storing and manually labeling large amounts of in-flight imagery. We combine the state-of-the-art classification and detection methods for filtering images with any bird occurrence and detecting the bird instances correspondingly. In this paper, our main focus is to provide some insights of deep learning algorithms to address issues of data processing in wildlife surveys.

[R2. Multiple annotation for the imagery]
In this paper, we explore such prototype models for automating data processing and do not include multiple annotations for the imagery. But this is a good suggestion and we are in agreement regarding the utility of this approach. We are developing an operational annotation and training data development workflow in which at least 2 annotators label the same set of imagery, with a process for arbitrating annotation discrepancies through a third species expert. This will allow us to better assess and control annotation data quality.

[R2. Cross-validating Mask R-CNN]
We agree that this is a good suggestion. We updated the cross-validation experiments in the manuscript.

[R2. Probability threshold selection for filtering]
In this paper, we simply select the probability threshold based on the performance over 78 additional images from Lake Michigan. We have not yet determined the specific threshold that we will use operationally and realize that the threshold may vary among surveys given specific survey objectives. Operationally, our workflow will incorporate some sampling of the images which do not pass the filter to manually review them and determine evaluate errors of omission. In a related effort we are working to develop statistical population estimation frameworks that incorporate machine-learning derived outputs in a rigorous manner. Errors of commission, passing imagery with no targets to subsequent workflow stages, primarily affect the in-flight performance and computational burden, and are less of our concern.

[R2. Compare Mask R-CNN with/without a classifier for filtering]
In the first stage of image filtering, we do not consider the fine-grained categories of bird instances, but only focus on detecting any bird occurrence. We, therefore, disagree with the recommendation to compare Mask R-CNN with and without a classifier for image filtering. We do agree that it would be a good idea to compare Mask R-CNN with / without a classifier for the

third stage. In our future work, we could conduct comparison between two different settings: 1) detection using the Mask R-CNN without a classifier and categorization using additional classification model, 2) detection and categorization using the Mask R-CNN with a classifier.

[R2. Compute the deviation of the first stage]
If we are understanding the comment correctly, no we did not evaluate the effects of error in the first stage classifier(filter) downstream in individual detection or classification stages. Our primary concern is how machine-learning outputs and errors at each stage in our workflow propagate as ecologically meaningful uncertainty in wildlife population estimates. While outside the scope of this paper, we are, therefore, focused questions about how machine-learning processes and outputs integrate within standard statistical frameworks for wildlife population size estimation. We envision, and are investigating, series of linked submodels that propagate error at all stages of the workflow (annotation, filter, detection, classification) into ecologically interpretable estimates of uncertainty in population size.

We are appreciative of the editor's and reviewers' thoughtful comments.

Journal Requirements

[Description of required permits}
No permits were required for this work. All field data collection was conducted by federal employees acting within the scope of their employment, with agency approval, and in coordination with other agencies as appropriate.

[Data repository and availability statement]
We have established the repository and listed details in the Data Availability Statement. The data associated with this publication will be provided through the U.S. Geological Survey's ScienceBase Catalog. The persistent identifier for all relevant data will be https://doi.org/10.5066/P9CBZQV1.

[Concern that the base map in Fig. 1 may be copyrighted]
We confirm that fig 1 is an original composition without reliance on copyrighted material.

Best,
Authors
